# Patient mobility for cardiac problems: a risk-adjusted analysis in Italy

**DOI:** 10.1186/1472-6963-13-56

**Published:** 2013-02-12

**Authors:** Messina Gabriele, Forni Silvia, Collini Francesca, Quercioli Cecilia, Nante Nicola

**Affiliations:** 1 Department of Public Health, Health Services Research Laboratory, University of Siena, Via Aldo Moro, 2 Siena, 53100, Italy; 2 Tuscan Regional Health Agency, Via Pietro Dazzi, 1 Florence, 50141, Italy

**Keywords:** Patient mobility, Risk adjustment, Cross-border health care, Quasi market

## Abstract

**Background:**

The Italian National Health System was revised in the last 20 years, introducing new elements such as efficacy, efficiency and competitiveness. Devolution to regional authorities has created a quasi-market system where patients can choose the hospital in which to be treated. Patient mobility therefore becomes an indicator of perceived hospital quality and of financial flows between the regions of Italy. Previous studies analyzed patient mobility in general or by specific disease/diagnosis-related groups but there is a lack of research on the influence of severity of patient condition. The aim of the study was to describe patient mobility, crude and stratified by disease severity, in cardiac surgery units of three health areas (HAs) in Tuscany (Italy).

**Methods:**

In this retrospective observational study, data was gathered from hospital discharge records obtained from the Tuscan Regional Health Agency, Italy. The three HAs (HA1, HA2, HA3) recorded 25,017 planned hospitalizations in cardiac surgery units in the period 2001–2007. Patients were stratified in four All Patient Refined Diagnosis Related Group (APR-DRG) severity levels. Gandy’s nomogram was used to describe how HAs met health care demand and their capacity to attract patients. Cuzick’s test was used to identify significant differences in time trends.

**Results:**

Raw data showed that the HAs met their own local health care demand. Stratifying by APR-DRG severity, it emerged that capacity to meet local demand remained unchanged for zero-to-minor severity levels, but one HA was less able to meet demand for moderate severity levels or to attract patients from other HAs and Regions of Italy. In fact, HA3 showed a decrease in admissions of local residents.

**Conclusions:**

The study highlights important differences between the three HAs that were only revealed by severity stratification: unlike HA3, HA1 and HA2 seemed able to deal with local demand, even after severity stratification. Planners and researchers can benefit from risk stratification data, which provides more elements for correct comparisons and interventions. In the context of patient mobility, the present study is a step in that direction.

## Background

Quality based on health outcomes instead of material goods and services is difficult to measure and compare [1]. Direct perceived quality is onerous and lengthy to assess, so objective indirect indicators allowing comparisons are preferred. Patient mobility, driven by patient preference, for one hospital over another, is an indirect indicator of perception of hospital quality. It is simple and objective: patients vote with their feet by changing hospital [2,3]. It is difficult to standardize patients and to make assumptions about a “typical patient”. Different patients make different choices in different situations [4]. Patients’ choices depend on their cultural and economic characteristics, their previous healthcare experiences, the reputation of the hospital staff and the network of relationships between the patient, hospital doctors and general practitioners/reference specialists. Most patients do not make completely rational choices [4,5].

However, assuming that patient choice depends at least partly on the perceived quality of services [2,6-8], analysis of patient preferences provides an idea of hospital quality as well as qualitative and quantitative lack of services [3,6,9-12]. Good perceived quality decreases escapes and attracts new patients.

At European level, research into patient mobility has increased significantly [2,13-15] due to EU regulations that allow patients to seek care anywhere in the Union [6]. Many papers have examined the factors influencing patient mobility [3] in different settings and countries [2,5,6,12,16-21]. Other studies have considered patient mobility under specific or general conditions [22-24], using data directly related to money flows or diagnosis-related groups (DRGs) [25,26]. However, to our knowledge, there has been no research combining patient mobility flow with disease severity. Severity could be an important determinant of patient mobility together with urgency of treatment. Patients who are aware or informed by doctors of their diagnosis probably choose in relation to disease severity. Patients with less severe conditions are unlikely to go far for treatment, especially if they feel that the local hospital can treat them, whereas patients are more likely to move, spontaneously or as suggested by their general practitioners, if they feel that local health services/doctors are unable to properly handle the situation.

The heart surgery sector is considered a marker of health service quality and has been the subject of national and international attention for more than two decades [27]. In Italy, it has been analyzed to determine whether the current organization of services cares for patients efficiently, effectively and without delay [28]. The aim of the present research was to describe patient mobility between cardiac surgery units, stratifying by disease severity in order to: i) acquire a better description of patient flows; ii) highlight patterns of patient flows hidden in crude data; iii) enable better understanding of critical areas where loss of quality is possible.

## Methods

### The Italian health system

The Italian National Health System, based on the Beveridge model of public funding and provision [29], recently celebrated its 34th anniversary. Before the reforms of the 1990s, citizens were free to choose where they were treated, and hospital funding was based on reimbursement of expenses incurred. Due to increased health costs, in 1992 the Italian Government passed a new law establishing a “quasi market” to improve efficiency. The state monopoly without any competition was replaced by a network of competitive independent non-profit providers, with the aim of encouraging competition between hospitals [30]. The universal system based on Beveridge model principles of the welfare state, in which citizens are free to choose where they are treated, was consolidated. This reform, which was implemented in 1995, also changed hospital funding to a system of prospective payment, with activity defined by diagnosis-related groups (DRGs) [31]. If a patient chooses to be treated in a hospital not managed by his/her local health unit (LHU), the patient’s LHU pays the hospital where the patient is admitted, anywhere in Italy.

### The setting

Tuscany, one of the twenty Regions of Italy, had a population of about 3,500,000 in the period 2001–2007, when 22% of the population was over 65 years of age. The region has 12 LHUs and 44 hospitals. Tuscan LHUs belong to three health areas (HA1, HA2, HA3), covering 4845, 6588 and 11561 km^2^ with population densities of 319, 189 and 70 persons/ km^2^, respectively. Every LHU has at least one hospital for general care. Major and specialist care is available at HA level in university hospitals, one per HA. The three Tuscan HAs have five cardiac units (two in HA1, two in HA2, one in HA3). All can treat patients requiring coronary artery bypass graft surgery (BPAC), aortic surgery, and heart valve repair or replacement. They perform more than 500 BPACs per year, a proxy volume indicator of good clinical competence [28]. All three HAs can treat any degree of severity.

The three HAs share borders, and are well connected to each other and with other Italian Regions by highways. Train connections are possible in all three HAs. Airports with national and international flights are only found in HA1 and HA2. Table 1 shows traveling times by car (minimum-maximum range) from the HAs to cardiac surgery units.

**Table 1:** Traveling time (minutes) by car (minimum-maximum range) from three Tuscan health areas to cardic surgery wards

	**Cardic surgery ward**				
		**HA1**	**HA2**	**HA3**	**Other -Out Region**
Health Area	HA1	10-60	30-150	30-150	>60
	HA2	30-150	10-60	60-210	>90
	HA3	30-150	60-210	10-90	>90

### Source of data

Pre-aggregate data, suitable for studying patient mobility, was gathered from hospital discharge records held by the Tuscan Regional Health Agency. The Agency filtered the data using inclusion criteria (see below) and calculated All Patient Refined Diagnosis Related Group (APR-DRG) severity levels. In Italy hospital discharge records are filed by public hospitals or hospitals sponsored by the National Health System. Hospital discharge records are medical-legal documents belonging to the hospital records of patients. They summarise administrative, clinical and personal data such as personal identification number, gender, planned/ordinary/emergency admission, date of birth, hospital department, discharge condition (alive/deceased), national hospital code, place of residence and DRG (used for financial purposes). The main condition treated or investigated during the relevant episode is defined by the International Classification of Diseases ninth revision Clinical Modification (ICD-9-CM). National hospital codes and place of residence are also important for determining regional compensation for patient mobility.

### Study design and population

This is a descriptive study investigating patient mobility between the three main health areas of Tuscany (Italy) in 2001–2007. The study population consisted of: i) patients resident in Tuscany or other Regions, admitted to Tuscan cardiac surgery units, and ii) Tuscan residents admitted to cardiac surgery units in other Italian Regions. Inclusion criteria for the population were: i) ordinary admissions (for acute pathologies and/or health problems that needed diagnosis and/or treatment) and planned admissions (for planned operations or other treatments); emergency admissions were excluded; ii) age > 16 years; iii) admission or transfer to pediatric or adult cardiac surgery units; iv) diseases and disorders included in Major Diagnostic Category 5 - Circulatory System. Table 2 shows the codes used to identify the cardiac surgery and selection procedures for the study population. Severity was calculated using All Patient Refined Diagnosis Related Groups (APR-DRGs) version 20 by 3M. The APR-DRG is an iso-severity classification system, a risk adjustment tool that stratifies patients at discharge on the basis of their severity levels. The severity stratification is calculated using information gathered from the hospital discharge records, including physiological alteration or loss of organ function; the levels were 0 = zero-minor; 1 = moderate; 2 = high; 3 = extreme [32,33].

**Table 2:** Codes used to identify the cardiac surgery and selection procedures for the study population

***Description***	***ICD 9 CM codes***
**By-pass**	36.03; 36.10 - 36.17; 36.19; 36.01 - 36.02; 36.05
**Other interventions for revascularization**	36.04; 36.09; 36.2; 36.3
**Operations on valves**	35.10 - 35.14; 35.20 - 35.28; 35.33; 35.00 - 35.04; 35.99
**Operations on the aorta**	38.34; 38.45; 39.54
**Heart Transplant**	37.51
**Other main procedures**	35.41 - 35.42; 35.50 - 35.54; 35.61 - 35.63; 35.70 - 35.72; 35.31; 35.32; 35.34; 35.35; 35.39; 35.9; 35.98; 36.91; 36.99; 37.10 - 37.12; 37.31 - 37.33; 37.35; 37.4; 37.94; 37.99; 38.04 - 38.05; 38.14 - 38.15; 38.35; 38.41; 38.65; 38.85; 39.21 - 39.23; 39.52; 39.57; 39.59
**Procedures associated**	34.03; 34.91; 37.00; 37.21 - 37.23; 37.26; 37.61-37.62; 37.70; 37.72; 37.78; 37.80; 37.83; 37.85; 37.87; 37.95; 38.91; 38.93; 39.61; 88.42 - 88.44; 88.50; 88.52 - 88.57
**Other diagnostic or therapeutic procedures on the heart**	37.24 - 37.25; 37.29; 37.63 - 37.64; 37.71; 37.73 - 37.77; 37.79; 37.81 - 37.82; 37.84; 37.86; 37.88 - 37.89; 37.91; 37.93; 37.98
**Other operations on vessels**	38.06 - 38.08; 38.10 - 38.13; 38.16 - 38.18; 38.36 - 38.40; 38.42 - 38.44; 38.46 - 38.48; 38.50; 38.53; 38.55; 38.59; 38.60 - 38.64; 38.66 - 38.69; 38.7; 38.80 - 38.84; 38.92; 38.94 - 38.99; 39.0; 39.1; 39.24 - 39.31; 39.41 - 39.51; 39.53; 39.55 - 39.56; 39.58; 39.60 - 39.66; 39.7; 39.71; 39.79; 39.8; 39.91 - 39.99
**Accessory procedures**	88.72 - 88.73; 88.92; 89.37; 89.43; 89.45; 89.43; 89.45; 89.50 - 89.52; 89.61; 89.64 - 89.65

The study population was classified as follows: i) patients admitted to hospital in their HA of residence (recovery of resident = RR); ii) patients admitted to hospital outside their HA of residence (escape = E), that is, a hospital of another HA in Tuscany or of another region of Italy; iii) patients admitted to a hospital of one HA coming from another HA or another Italian Region (attraction = A).

### The Gandy nomogram

Analysis of patient flows was undertaken using Gandy’s nomogram [34-38], a useful and flexible method, requiring minimal data, for comparing access across many geographical areas in one presentation. Managers and analysts can use it to explore problematical issues relating to access to public services in a wide range of situations and sectors. It can be used for exploratory data analysis and benchmarking for decision-makers. In the early stages of an investigation, it is useful to indicate where more detailed, localized analysis may be required, and to support decisions [37]. By design, it focuses on percentages, however because percentages mask relative size, we made sure that the percentages produced could be compared. The public services in which the nomogram is most useful for describing access are those with area perspective and mobility is a key factor.

The nomogram displays changes in patient flows using repeated time investigations and its three variables:

RR (Hospitalisation of inhabitants in their own area of residence);

A (Hospitalisation of “immigrants” from other areas: attracted patients);

E (Hospitalisation of residents of the local area in other facilities: escaping patients). RR+A expresses the number of patients admitted to a HA, irrespective of where they reside (hospital activity), and RR+E expresses patients residing in a HA admitted to any hospital (demand for healthcare). The X Axis = RR/(RR+A)*100 expresses the percentage of total admissions of local residents to the HA. The Y Axis = RR/(RR+E)*100 expresses the percentage of local residents’ demand for hospitalisation satisfied in a HA.

Attaction power increases from right to left along the “x” axis, while escapes increase from the upper to the lower part of the “y” axis. Equivalent balanced migratory flow, A=E, is indicated by the diagonal to the point W where A=E=0, i.e. there is no migration from other HAs or to other facilities.

In the quasi market scenario of the Italian Health System it is important: i) to meet the demand of the citizens of an area (LHU, HA or Region) - upper right quadrant of the nomogram (RR>A and E); ii) to attract patients and to limit escapes - points above the diagonal, dividing the upper right quadrant (A > E). Points in other quadrants are considered less than optimal because most hospitals/HAs are non profit. Public hospitals’ first objective is to meet the local demand of their populations for medical care and only second to attract patients from other areas. If the first objective is met, patients are kept and escapes avoided. The direction of movement depends on an area’s success in attracting and retaining patients. If an area increases admissions from outside its borders, then its position moves progressively into the upper left-hand quadrant. Conversely, if it is failing to exploit opportunities, then its position moves progressively into the bottom right quadrant.

Gandy nomograms were designed for admissions to cardiac surgery units with and without APR-DRG stratification. Significant variations in flow trends were assessed using the Cuzick test [39], a Wilcoxon-type test for trend across a group of three or more independent random samples, in which the assumptions are that i) the data must be at least ordinal and ii) groups must be selected in a meaningful order (i.e. ordered). Statistical significance was set at P < 0.05.

## Results

Between 2001 and 2007, 23,645 Tuscan residents were admitted to Italian cardiac surgery units. The five Tuscan cardiac units had 25,017 admissions in the same period. Twenty-seven percent of the 23,645 Tuscan patients were escapes with similar percentages in the three HAs. Attractions accounted for one third of Regional admissions. Attraction from other Regions occurred mainly in HA2 (43.4%), followed by HA1 (30.3%) and HA3 (26.3%). Mean time to discharge ranged from 10.9 days (standard deviation 0.5) in HA1 to 12.9 days (standard deviation 0.6) in HA2 and 12.5 days (standard deviation 0.6) in HA3.

**Figure 1:**
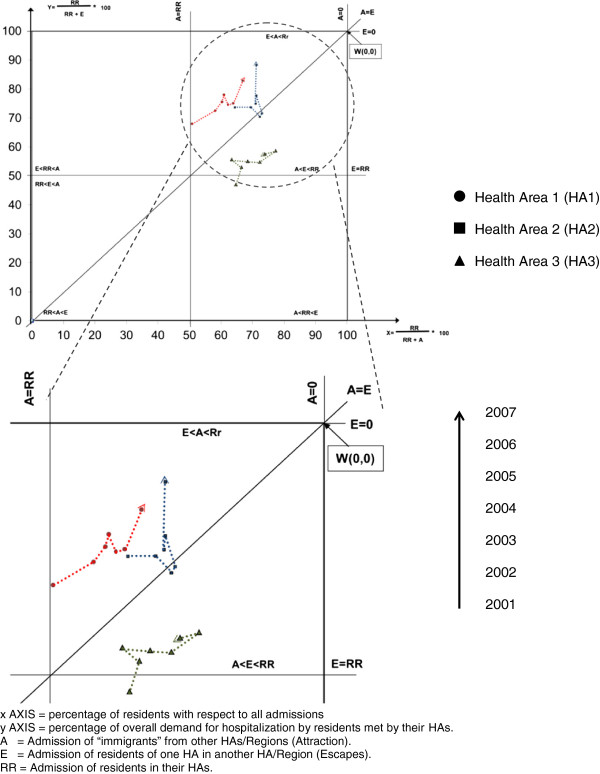
Gandy nomogram for cardiac units.

Crude analysis showed that all HAs were in the upper right quadrant of the nomogram, although HA3 was below the other two (more escapes and fewer attractions). Over the 7-year period, all HAs showed a reduction in attractions and escapes: points in the nomogram moved from left to right and upwards to different degrees (Figure 1). Reduction trends were significant for escapes and attractions in HA1 (E: p=0.029; A: p=0.029, respectively) and HA3 (E: p=0.018; A: p=0.031, respectively).

**Figure 2:**
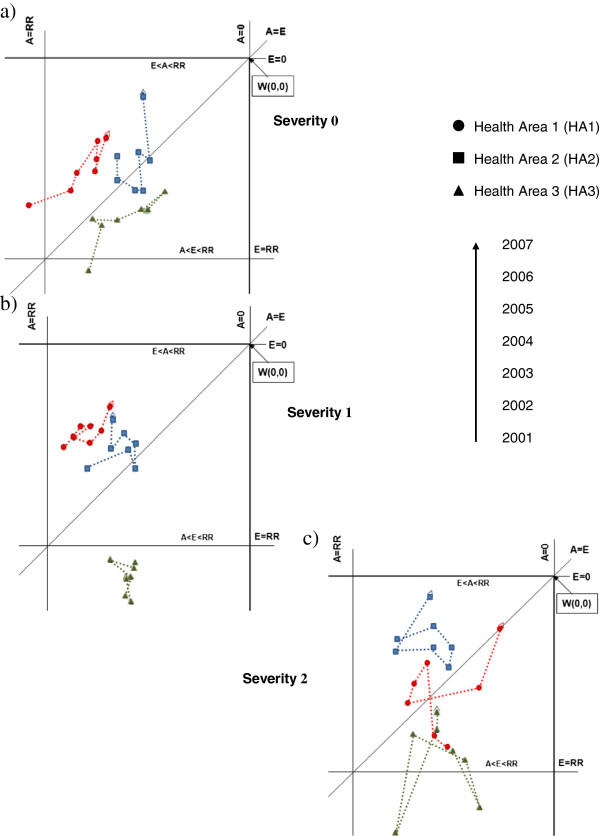
Gandy nomograms for cardiac surgery units by APR-DRG severity level.

In stratifying for the four APR-DRG severity levels, we omitted the fourth (most severe) level because it contained too few cases. Regarding the zero-minor severity level (0), all HAs were in the upper right quadrant of the nomogram and showed a decreasing trend in attractions and escapes over the study period. Health Areas 1 and 2 maintained a higher position than HA 3 (Figure 2a). At the moderate severity level (1), HA1 and HA2 maintained their positions and trends in time, whereas HA3 passed into the lower right quadrant of the nomogram, indicating more escapes than resident admissions and attractions. The time trend indicated an increase in escapes (Figure 2b). At the high severity level (2), HA2 showed the best results, improving its position with respect to HA1, reducing escapes and maintaining good attraction power. HA3 seemed to resist escapes and maintained its attraction (Figure 2c).

## Discussion

Many variables may play a role in patient mobility: distance, type of hospital/unit, expected outcome, costs, medical staff acquaintances, advice from physicians or friends, previous experiences and waiting lists [3-6,12,20,23,24,40]. Our research was based on the assumption that patient mobility is partly influenced by active choice.

It is easy to determine that patient mobility is influenced by active choice on the part of a patient, possibly guided by his/her GP, when the hospital is distant from the patient’s home. For example, studies have highlighted distances of more than 1000 km covered by patients living in southern Italy hospitalised in the north [5,23]. Another form of mobility, termed “physiological” [5], is influenced less by active choice than by reasons unrelated to the quality of care. Such a reason is geographical position: in border-areas it may be rational for patients to access cross-border care if this implies shorter distances [2]. In this case, crossing the border of the LHA may express a preference dictated by easy access rather than by better care. In some cases patients move without making any rational choice [4], whereas in others, when patient condition is at least moderately severe or complex [5,25], patients are driven by their own active choices or by those of their referring GPs. In the present study, the Gandy nomogram, trends and APR-DRG severity stratification described general aspects of patient mobility and also identified some unexpected situations.

The main findings of the study were as follows:

i) Movement of the points from left to right in non stratified nomograms showed that all HAs were losing capacity to attract patients and gaining capacity to avoid escapes. The trends showed that HA1 and HA2 succeeded in regaining lost patients, whereas HA3, despite a positive trend, was slow in doing so. Patient trust in health services may have important repercussions on future trends. Retaining/attracting patients takes time and persistence, while loss and distrust of patients have major consequences.

ii) Aspects embedded in the phenomenon of patient mobility emerged with severity stratification. Trend analysis highlighted that while all HAs showed similar trends of unstratified mobility at the zero-minor severity level, at the moderate level HA1 maintained its position (top upper right quadrant), HA2 had more escapes than attractions (lower part of upper right quadrant) and HA3 descended into the sector where escapes are more numerous than attractions and the return of residents. Stratifying at the moderate severity level, HA1 and HA2 maintained or improved their position over the years, especially in recovering escapees. This aspect is particularly important because it shows that patients could tend to remain in the same HA even when their condition is moderately severe, probably demonstrating trust in the health services where they live. Health Area 3 had all its points in the lower part of the upper right quadrant of the nomogram and in the quadrant where escapes are more numerous than attractions and recovery of escapees. The numbers were few and therefore percentages more likely to vary in position, and no trend was detected. The broad cloud of points may indicate a will to improve position, though it is not easy to return to the upper quadrants.

While extreme severity should limit patient mobility because of the objective difficulty of moving critical patients (points should be in the middle of the upper right quadrant), when patients do indeed move of their own initiative, it is very informative. In seeking medical care far from home and family, in a different environment, and paying all associated expenses [2,12,23], the patient votes twice, expressing distrust of the service available locally and belief in obtaining better treatment elsewhere. For the hospital, this patient’s choice is a double failure, indicating loss of patient trust and a lost opportunity to treat the patient, with negative economic consequences as well. The stratified nomograms provided a description that was not possible with the unstratified data. Differences in hospital selection exist and are influenced by disease severity. Moreover, trend analysis showed important changes underway: patients have modified their preferences for different hospitals. Whether these changes are influenced by the real or perceived quality of care is relevant to what measures should be taken. If there is an objective lack of service/care, specific improvements are necessary to limit escapes. If the changes are only driven by patient perception, managers should improve communication with the public and general practitioners [41]. Involvement of the latter is particularly important in Italy where GPs are patients’ first contact with the health system. Since general practitioners’ advice may influence patient choice [20,40] it is necessary that GPs be informed and kept up to date on LHU services. Health planners should ensure equal distribution and access of services to patients: the escape of patients could highlight a problem in achieving this aim.

iii) Comparing unstratified with severity-stratified data, it emerged that it was easier to maintain a position in the nomogram if the points were in upper part of the top right quadrant than in the lower part. The points of HA1 and HA2 generally maintained their positions, irrespective of APR-DRG stratification, whereas HA3 did not. An important difference revealed by stratified analysis was that as severity increased, the possibility of retaining patients decreased. Patients move to other areas when they feel that local health services/doctors are unable to properly care for them.

This descriptive study was conducted to acquire knowledge in an increasingly important field of public health. Stratification is always needed when comparisons are made, as we found in our study. Patient mobility also calls for severity stratification, although in the present case it was already partially selected and regarded a specific disease group. The APR-DRG system seems to identify some critical aspects, highlighted by the stratifications, and to provide useful details for investigating patient healthcare choices at different severity levels.

The differences identified studying cardiac surgery admissions are likely to exist for other diseases as well, but further studies are necessary. Other risk adjustment tools, such as the Charlson [42,43] or the Elixhauser Comorbidity Index [44], which use hospital discharge records as a basis for determining severity scores, could be used to verify our findings and to conduct sensitivity analysis [45,46]. For example, the APR-DRG produced very few cases at the extreme (severest) level (3), which prompted us to discard this small sample.

On one hand, risk adjustment tools do not cover all the possible aspects of confounders: they are not definitive in assessing borderline differences. Although widely used and improving in time, hospital discharge records only contain what are considered to be major health conditions. On the other hand, risk adjustment tools can provide useful indications if differences are large, as we have seen with stratification and trend.

The study has certain limits: i) It does not quantify “physiological mobility”, that is, movements from areas near Regional/HA borders, unrelated to improvements in heath care quality [5], but due to closeness/easy access to hospitals in other areas (Tuscan HAs and neighbouring Regions of Italy). Physiological mobility is more evident in neighbouring areas. ii) Other factors, not detected by this study, could have influenced patient mobility. For example, hospitals may influence the referral pattern of GPs. In our study this possibility should be limited, because the hospitals/HAs had similar characteristics and offered similar services. It is more likely to occur when differences exist. The fact remains that GPs may refer patients to one hospital or another for various reasons, such as: locality and convenience, familiarity with hospital, good overall service, sub-specialist care, clinical need of patient, waiting time for appointment, good communication at hospital or a known consultant [47]. iii) It may attribute patient mobility to citizens officially resident in areas different from those in which they actually live [48-50]. Such mobility is not easy to detect and is difficult to measure; it may also be influenced by several factors. A previous attempt to estimate this portion of mobility [49] assumed that it could be equivalent to: i) mobility for diseases without complications, treatable in any hospital and ii) emergency admissions where patients cannot choose [38].

## Conclusions

This research shows that patient mobility is influenced by the severity of their medical condition. Patients move with their feet, and they move differently in relation to disease severity. Planners and researchers can benefit from risk-adjusted data because it provides a better description of patient mobility preferences. Stratification/standardization is essential for correct comparisons and interventions. The present study is a step in that direction but further research is needed. *Ad hoc* studies for identifying and weighing the causes of patient mobility are important. Together with descriptive studies, such as the present one, they will enable better understanding of the phenomenon of patient mobility.

## Ethics committee approval

The authors declare that ethical approval for conducting the research was not needed. Data, administrative ones, aggregated in the form in which were studied, were anonymous and did not allow patients identification.

## Consent

For the publication of this report it was not necessary written consent of patients because the data, administrative ones, aggregated in the form in which were studied, were anonymous and did not allow patients identification.

## Competing interests

The authors declare that they have no conflicts of interest.

## Authors’ contributions

Regarding their various contributions, the authors declare that GM had the idea for the article, performed the literature research, collaborated in performing the study, carried out the data analysis and wrote the article, SF collected the data, collaborated in performing the study and reviewed drafts of the manuscript, FC collected the data, collaborated in performing the study and reviewed drafts of the manuscript, CQ collaborated in data analysis, interpretation, writing the article, conceptualization of ideas and reviewed drafts of the manuscripts, and NN collaborated in performing the study, supervised the work, helped conceptualize ideas and reviewed drafts of the manuscripts. All authors contributed to conception of the article, drafting and revision of content. All authors read and approved the final manuscript.

## Pre-publication history

The pre-publication history for this paper can be accessed here:

http://www.biomedcentral.com/1472-6963/13/56/prepub
